# Multisectoral action and community involvement are required for long-term improvements in public health

**DOI:** 10.5588/pha.24.0022

**Published:** 2024-06-01

**Authors:** G.N. Kazi, H.D. Blackbourn

**Affiliations:** The International Union Against Tuberculosis and Lung Disease (The Union), Paris, France.

**Keywords:** sustainable development goals, SDG, tuberculosis, PHC, social determinants of health, SDH

Public Health Action (PHA) has been the flagship journal for operational research for more than a decade.^1,2^ Since its launch, the journal’s coverage has adapted in line with the broader global health agenda, including the United Nations Sustainable Development Goals (SDGs).^3^ SDG 3 is one of the 17 SDGs and focuses on ensuring good health and promoting well-being at all stages of the life cycle.^4,5^ The goal has 13 targets and 28 indicators including progress towards reducing maternal mortality, ending all preventable deaths under five years of age, and prevention and control of communicable and noncommunicable diseases. Progress is also measured on a range of diverse issues such as tobacco control, developing and providing universal access to vaccines and medicines, and developing early warning systems for global health risks. The process involves ending the epidemics of AIDS, TB, malaria, and other communicable diseases. SDG 3 is highly aspirational in its aims to achieve universal health coverage and equitable access to healthcare services. Embedded in the UN’s 2030 Agenda is also the removal of health and social inequalities, the effects of climate change, and the imperative for pandemic preparedness in a world still reeling from the effects of COVID-19.^6^

If nothing else, the SDGs illustrate the substantive problems that we must address to achieve progress in healthcare. We believe that it is unrealistic to expect significant improvements to health and well-being for all unless progress is made on the targets for SDG-3. In addition, an individual’s health is influenced by many non-medical factors, termed the social determinants of health (SDH). These include where we are born and whether we have access to money, power, or resources at the local and national levels. For people born in low- and middle-income countries (LMICs), or born into poverty within high-income countries, significant improvements to health depend on the synergies and complementarities between SDG-3 and the other SDGs.^7–9^ It is therefore essential that we also make progress in ending poverty (SDG-1), improving access to education (SDG-4), reducing inequalities (SDG-10), provide food security (SDG-2), provision of water and sanitation (SDG-6) and mitigating climate change (SDG-13).

Developing linkages between these SDGs will also address two critical gaps in primary healthcare (PHC), namely the lack of community involvement and multisectoral action.^10–12^ These are significant problems for weak healthcare systems in LMICs. A recent Editorial in PHA highlighted the importance of Community Connect at the Union’s World Conference on Lung Health.^11^ This forum recognises the importance of communities and leverages their strengths to make meaningful progress on the End-TB Strategy. There is also a need to enhance collaboration between other sectors (e.g., housing, environment and economy) and groups (e.g., government, civil society and the private sector).^14^ By engaging all relevant parties, various stakeholders can combine their knowledge, expertise and resources with the common goal of achieving a healthier and more productive society. An undertaking of this magnitude requires political commitment at the highest level within national governments and international agencies. Naturally, this commitment includes the need to provide sustained financing for efficient implementation and positive outcomes.
